# Atypical Presentation of Classical Hodgkin Lymphoma as Superior Vena Cava Syndrome With Paraneoplastic Eosinophilia and Rash: A Case Report

**DOI:** 10.7759/cureus.109943

**Published:** 2026-05-30

**Authors:** Pragnesh D Patel, Sowmya Yamarthy, Devon Thorpe, Julianne Esguerra, Ashish Shah

**Affiliations:** 1 Internal Medicine, Overlook Medical Center, Summit, USA; 2 Hematology and Oncology, Overlook Medical Center, Summit, USA

**Keywords:** eosinophilia, hodgkin lymphoma, mediastinal mass, paraneoplastic syndrome, pruritus, superior vena cava syndrome

## Abstract

A 47-year-old female presented with progressive left-sided facial swelling, exertional dyspnea, and a generalized pruritic rash. Physical examination revealed significant facial edema, cervical lymphadenopathy, and a diffuse maculopapular eruption. Laboratory evaluation demonstrated an absolute eosinophil count of 1.81 x 10^9^/L. Cross-sectional imaging with a computed tomography (CT) scan identified a large anterior mediastinal mass invading the superior vena cava (SVC) and left brachiocephalic vein, supporting the diagnosis of SVC syndrome. Histopathologic evaluation of an ultrasound-guided core cervical lymph node biopsy confirmed classical Hodgkin lymphoma (cHL), nodular sclerosis subtype. This case highlights an uncommon initial presentation of cHL characterized by the convergence of SVC syndrome, eosinophilia, and a paraneoplastic pruritic rash in the absence of classic B symptoms. Prompt identification of these atypical manifestations must be reconciled with the broader clinical context to avoid delays in diagnosis and treatment. Furthermore, this case emphasizes the necessity of obtaining a definitive tissue diagnosis before initiating corticosteroid therapy, which can mask lymphoproliferative architecture and obscure pathologic findings.

## Introduction

Classical Hodgkin lymphoma (cHL) most commonly presents with painless lymphadenopathy and constitutional *B symptoms*, including fever, night sweats, and weight loss. However, less common presentations may delay recognition. Superior vena cava (SVC) syndrome is most frequently caused by lung cancer, while lymphoma accounts for a smaller proportion of cases [1,2]. Although nodular sclerosis Hodgkin lymphoma commonly involves the mediastinum, progression to clinically apparent SVC syndrome remains rare, with fewer than 30 reported cases in the literature as of 2021 [1-4].

Paraneoplastic manifestations may further obscure the diagnosis. Peripheral eosinophilia and pruritic or inflammatory cutaneous eruptions are recognized features of cHL and may precede diagnosis [5-7]. These dermatologic findings are often nonspecific and may be misattributed to allergic or inflammatory conditions, contributing to diagnostic delay [5,6]. We present a diagnostically challenging case of cHL manifesting as SVC syndrome with marked eosinophilia and a pruritic rash in the absence of classic systemic symptoms.

## Case presentation

A 47-year-old female with a medical history of hyperlipidemia, gastritis, and asthma presented with three months of progressive left-sided facial swelling and exertional dyspnea. Symptoms began with facial discomfort and trismus, followed by the development of a generalized pruritic, brown, maculopapular rash involving the torso and proximal extremities. She also noted a newly palpable right supraclavicular mass but denied fever, night sweats, or unintentional weight loss.

On examination, the patient was afebrile and hemodynamically stable. Physical findings included left facial edema, a firm right supraclavicular lymph node (~3 cm), left submandibular lymphadenopathy, and a diffuse maculopapular rash over the abdomen and upper extremities.

Laboratory evaluation revealed marked eosinophilia with an absolute eosinophil count of 1.50 x 10^9^/L. The complete blood count demonstrated mild normocytic anemia with a hemoglobin of 12 g/dL. White blood cell and platelet counts were both within normal limits on presentation. Iron studies (including serum iron and ferritin), vitamin B12, and folate levels were within normal limits, making a nutritional etiology for the anemia less likely. Lactate dehydrogenase was elevated, consistent with increased cell turnover. Renal and hepatic function tests were within normal limits, confirming preserved end-organ function at presentation. Collectively, these laboratory findings supported a systemic inflammatory or malignant process and directed subsequent diagnostic decisions (Table 1).

**Table 1:** Key laboratory findings on presentation. MCV, mean corpuscular volume

Laboratory test	Value	Reference range
WBC	7.34 × 10⁹/L	4.0-11.0
Hemoglobin	12.0 g/dL	12.0-16.0
MCV	87.0 fL	80-100
Platelets	291 × 10⁹/L	150-400
Absolute eosinophils	1.50 × 10⁹/L	<0.50
Eosinophils (%)	20.4%	0-6
Absolute lymphocytes	0.63 × 10⁹/L	1.0-3.5
LDH	254 U/L	140-280
Albumin	3.3 g/dL	3.5-5.0
Creatinine	0.85 mg/dL	0.6-1.1
AST	15 U/L	10-40
ALT	20 U/L	7-56
Serum iron	56 µg/dL	60-170
Ferritin	145 ng/mL	13-150
Vitamin B12	486 pg/mL	200-900
Folate	10.3 ng/mL	>4.0

Initial contrast-enhanced CT of the neck and chest demonstrated a large, loculated soft-tissue mass centered in the right anterior mediastinum, measuring approximately 7.6 cm x 5.7 cm (Figure 1). On axial imaging, the mass occupied the perivascular space and extended across the midline with effacement of normal mediastinal planes, concerning for an invasive malignant process. The mass abutted and compressed the SVC adjacent to the ascending aorta (Figure 1), with loss of the vessel's usual rounded contour, suggesting impaired venous outflow. Coronal reformatted images demonstrated long-segment narrowing of the SVC along its mediastinal course as the vessel remained enveloped by the tumor over multiple contiguous segments (Figure 2), findings consistent with SVC syndrome.

**Figure 1:**
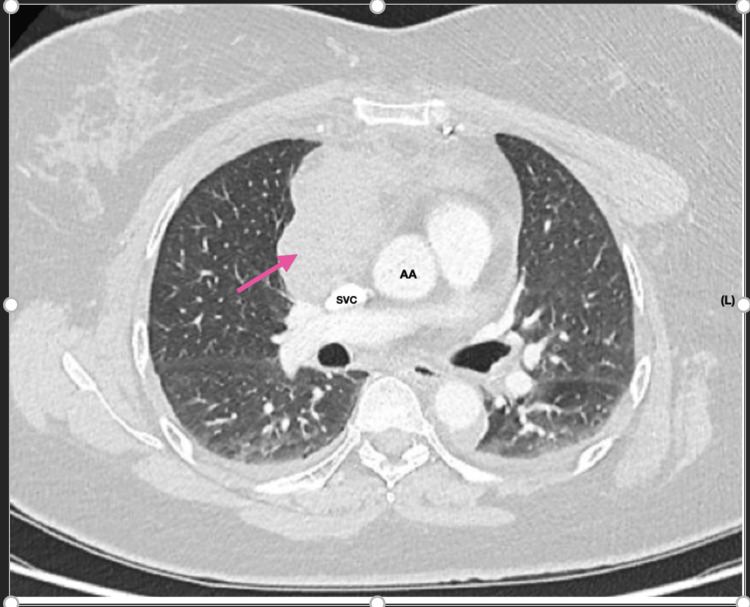
Axial contrast-enhanced computed tomography (CT) of the chest demonstrating a large anterior mediastinal soft-tissue mass (pink arrow) abutting and compressing the superior vena cava (SVC), which appears narrowed relative to the adjacent patent ascending aorta (AA), consistent with SVC syndrome.

**Figure 2:**
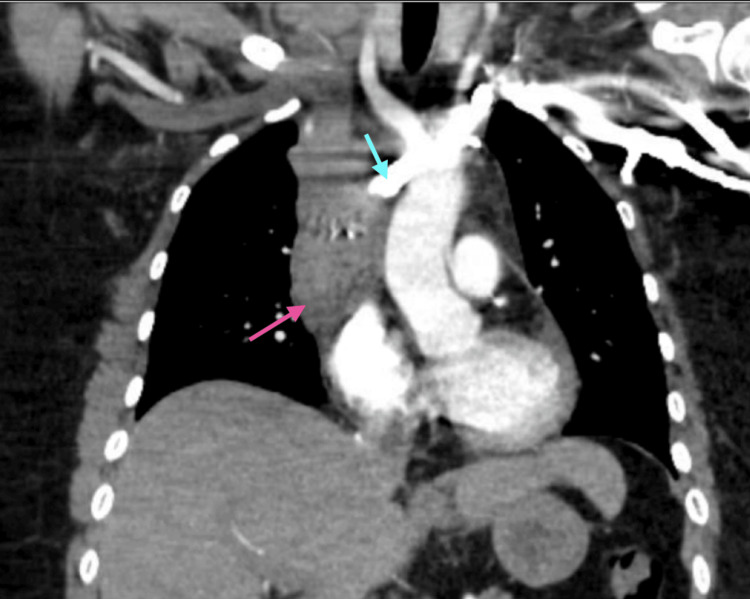
Coronal contrast-enhanced computed tomography (CT) angiogram of the chest demonstrating a large anterior mediastinal mass (pink arrow) causing marked extrinsic compression and narrowing of the superior vena cava at the confluence of the brachiocephalic veins (blue arrow), with associated mass effect on adjacent mediastinal structures, consistent with superior vena cava syndrome.

Imaging demonstrated thrombosis of the right internal jugular vein, likely secondary to impaired central venous drainage from SVC obstruction. Extensive mediastinal, supraclavicular, and cervical lymphadenopathy was present. No distant disease was identified on abdominal or pelvic imaging.

Taken together, these imaging findings were concerning for an aggressive anterior mediastinal neoplasm with extensive nodal involvement, significant encasement and narrowing of the SVC, and associated right internal jugular vein thrombosis (Figures 1, 2).

Hematology was consulted early in the hospital course. As the patient remained hemodynamically stable without signs of airway compromise, corticosteroids were deferred to avoid obscuring the histopathologic diagnosis, in accordance with guideline-based recommendations. Therapeutic anticoagulation with enoxaparin was initiated for the right internal jugular vein thrombosis.

An ultrasound-guided core biopsy of the right cervical lymph node demonstrated effaced nodal architecture with Reed-Sternberg cells and lacunar variants in a fibrotic background. Though flow cytometry was negative, immunohistochemistry showed CD30 positivity, dim PAX5 expression, and an absence of CD20, confirming nodular sclerosis classical Hodgkin lymphoma (Figures 3, 4).

**Figure 3:**
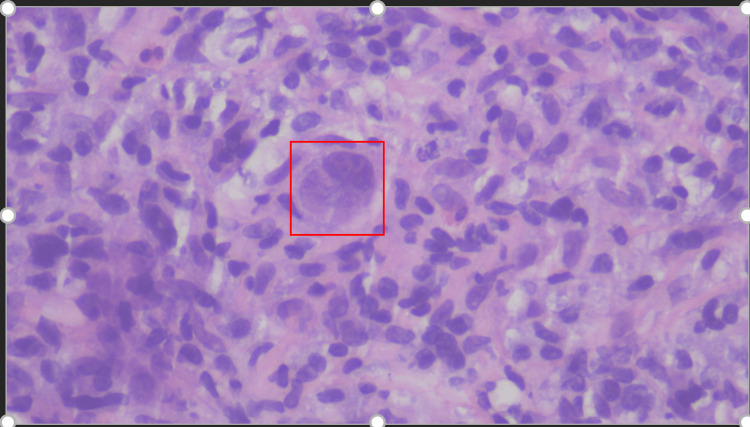
Hematoxylin and eosin (H&E) stain at 40× magnification demonstrating a classic Reed-Sternberg cell within a mixed inflammatory milieu, supporting the diagnosis of classical Hodgkin lymphoma.

**Figure 4:**
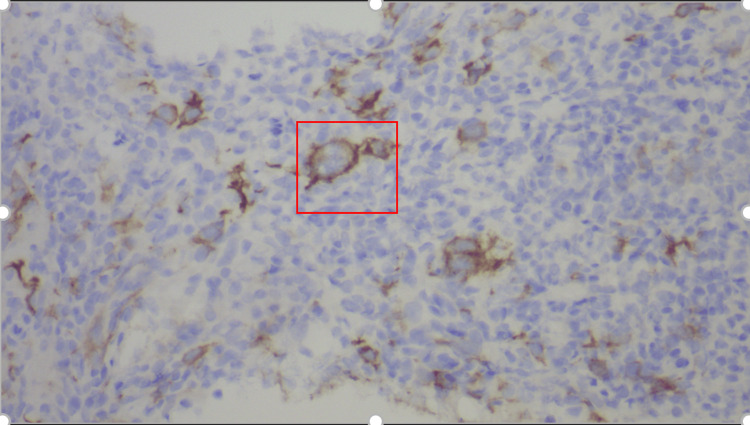
Immunohistochemical stain for CD30 at 40× magnification. The image demonstrates large, atypical Reed-Sternberg cells showing strong membranous and perinuclear (Golgi) positivity, outlined in the red box. These neoplastic cells are interspersed within a dense reactive background of small T lymphocytes and other inflammatory cells, confirming the diagnosis of classical Hodgkin lymphoma.

The patient’s rash partially improved with topical corticosteroids, possibly suggestive of an inflammatory component. She was discharged on apixaban with plans for outpatient positron emission tomography-computed tomography (PET-CT) staging and oncology follow-up. Unfortunately, the patient was subsequently lost to follow-up.

## Discussion

cHL most commonly presents with painless lymphadenopathy and constitutional *B symptoms*, including fever, night sweats, and weight loss. However, atypical presentations may obscure the diagnosis and delay appropriate management. This case highlights an unusual presentation of nodular sclerosis-type cHL characterized by SVC syndrome, marked eosinophilia, and a paraneoplastic pruritic rash in the absence of classic systemic symptoms.

SVC syndrome is characterized by facial swelling, dyspnea, and venous congestion; it most commonly arises from thoracic malignancies [2]. While lung cancer accounts for the majority of cases, lymphoma represents an important subset [3]. Nodular sclerosis cHL frequently involves the mediastinum, occurring in approximately 60-70% of cases and often presenting as bulky disease [1]. Despite this predilection, progression to clinically apparent SVC syndrome remains uncommon in Hodgkin lymphoma, underscoring the atypical nature of this presentation [3]. In the present case, the gradual onset of facial swelling, venous congestion, and exertional dyspnea reflected impaired venous return caused by extensive mediastinal lymphadenopathy with encasement of the brachiocephalic vein and SVC. Importantly, SVC syndrome related to lymphoma often evolves insidiously and is not immediately life-threatening, allowing time for diagnostic evaluation in clinically stable patients before empiric intervention [2].

An additional diagnostic challenge in this case was the presence of marked eosinophilia and a generalized pruritic rash. Peripheral eosinophilia is a recognized feature of cHL and is thought to result from cytokine-driven mechanisms within the tumor microenvironment, particularly interleukin-5 (IL-5) mediated eosinophil recruitment and survival [5]. Cutaneous manifestations of Hodgkin lymphoma range from generalized pruritus to eczematous or inflammatory eruptions and may precede diagnosis by weeks to months [6]. These manifestations are frequently nonspecific and may be misattributed to allergic or dermatologic conditions, contributing to diagnostic delay [6,7]. Prior reports describe pruritus, dermatitis, and inflammatory skin changes as paraneoplastic manifestations of Hodgkin lymphoma [7]. In this patient, partial improvement of the rash with topical corticosteroids suggested an inflammatory component; however, paraneoplastic cutaneous manifestations are often incompletely responsive to symptomatic therapy and tend to improve only with effective treatment of the underlying malignancy [6]. Recognition of eosinophilia and cutaneous symptoms as potential signs of lymphoma is therefore critical, particularly when accompanied by lymphadenopathy or other systemic abnormalities [5,6].

This case also illustrates key diagnostic principles in suspected Hodgkin lymphoma. Flow cytometry is frequently nondiagnostic because malignant Reed-Sternberg cells are sparse and embedded within a reactive inflammatory background [8]. As a result, definitive diagnosis requires adequate tissue sampling with histopathologic and immunohistochemical evaluation; fine-needle aspiration (FNA) alone is generally insufficient [9]. In this case, core biopsy demonstrated classic morphologic features and an immunophenotype consistent with nodular sclerosis cHL, including CD30 positivity, dim PAX5 expression, and an absence of CD20, underscoring the importance of correlating morphology with immunophenotyping for an accurate diagnosis [8,9].

The management of suspected malignancy-associated SVC syndrome requires careful coordination. Although corticosteroids may rapidly reduce tumor-associated edema and improve symptoms, their administration before tissue diagnosis can obscure histopathologic findings and should be avoided in clinically stable patients [2]. Accordingly, corticosteroids were deferred in this case until diagnostic sampling was completed. The presence of venous compression with suspected internal jugular vein thrombosis supported the initiation of therapeutic anticoagulation, recognizing the combined roles of mechanical obstruction, endothelial injury, and malignancy-associated hypercoagulability in thrombus formation [2]. Current American Society of Clinical Oncology (ASCO) guidelines recommend direct oral anticoagulants (DOACs) as options for the treatment of cancer-associated venous thromboembolism in appropriately selected patients, with careful consideration of bleeding risk and potential drug interactions [10,11].

Although staging studies were initiated and outpatient oncology follow-up was arranged, the patient was ultimately lost to follow-up, which prevented monitoring of response to therapy. This reflects a persistent challenge in cancer care delivery, as gaps in coordination and psychosocial barriers can delay or prevent the initiation of definitive therapy [12]. This issue is particularly consequential in Hodgkin lymphoma, a malignancy with high cure rates when modern, risk-adapted therapies are delivered in a timely fashion [13].

## Conclusions

This case illustrates an atypical presentation of cHL manifesting as SVC syndrome, paraneoplastic eosinophilia, and a pruritic rash. It highlights the necessity of considering lymphoma in the differential diagnosis of SVC syndrome, particularly when accompanied by systemic or dermatologic features. Early recognition of these paraneoplastic manifestations may facilitate a more rapid diagnosis.

Furthermore, this case emphasizes the importance of diagnostic sequencing; in clinically stable patients, prioritizing tissue confirmation before corticosteroid administration is essential to ensure histopathologic accuracy. Comprehensive management must also address associated complications, such as malignancy-associated venous thrombosis. Finally, the outcome of this case underscores the critical role of care coordination and longitudinal follow-up, as Hodgkin lymphoma remains a highly curable malignancy when treated promptly with contemporary therapeutic strategies.
